# Hydrolytic Amino Acids Employed as a Novel Organic Nitrogen Source for the Preparation of PGPF-Containing Bio-Organic Fertilizer for Plant Growth Promotion and Characterization of Substance Transformation during BOF Production

**DOI:** 10.1371/journal.pone.0149447

**Published:** 2016-03-14

**Authors:** Fengge Zhang, Xiaohui Meng, Chenglong Feng, Wei Ran, Guanghui Yu, Yingjun Zhang, Qirong Shen

**Affiliations:** 1 National Engineering Research Center for Organic-based Fertilizers, Nanjing Agricultural University, Nanjing, 210095, China; 2 Grassland& Environmental Engineering Lab, Nanjing Agricultural University, Nanjing, 210095, China; 3 Jiangsu Collaborative Innovation Center for Solid Organic Waste Utilization, Nanjing Agricultural University, Nanjing, 210095, China; Old Dominion Univ., UNITED STATES

## Abstract

Opportunity costs seriously limit the large-scale production of bio-organic fertilizers (BOFs) both in China and internationally. This study addresses the utilization of amino acids resulting from the acidic hydrolysis of pig corpses as organic nitrogen sources to increase the density of *Trichodermaharzianum*T-E5 (a typical plant growth-promoting fungi, PGPF). This results in a novel, economical, highly efficient and environmentally friendly BOF product. Fluorescence excitation-emission matrix (EEM) spectroscopy combined with fluorescence regional integration (FRI) was employed to monitor compost maturity levels, while pot experiments were utilized to test the effects of this novel BOF on plant growth. An optimization experiment, based on response surface methodologies (RSMs), showed that a maximum T-E5 population (3.72 × 10^8^ ITS copies g^−1^) was obtained from a mixture of 65.17% cattle manure compost (W/W), 19.33% maggot manure (W/W), 15.50% (V/W)hydrolytic amino acid solution and 4.69% (V/W) inoculum at 28.7°C after a 14 day secondary solid fermentation. Spectroscopy analysis revealed that the compost transformation process involved the degradation of protein-like substances and the formation of fulvic-like and humic-like substances. FRI parameters (P_I, n_, P_II, n_, P_III, n_ and P_V, n_) were used to characterize the degree of compost maturity. The BOF resulted in significantly higher increased chlorophyll content, shoot length, and shoot and root dry weights of three vegetables (cucumber, tomato and pepper) by 9.9%~22.4%, 22.9%~58.5%, 31.0%~84.9%, and 24.2%~34.1%, respectively. In summary, this study presents an operational means of increasing PGPF T-E5 populations in BOF to promote plant growth with a concomitant reduction in production cost. In addition, a BOF compost maturity assessment using fluorescence EEM spectroscopy and FRI ensured its safe field application.

## Introduction

*Trichoderma* species exhibit extremely high levels of ecological adaptability through their symbiotic colonization of plants and saprophytic existence in all soil types. Their capacity to improve plant growth and promote health in agricultural systems has been well documented. Recognized as plant growth-promoting fungi (PGPF) [1], *Trichoderma* form mutualistic relationships with many crops [2, 3]and have been widely implemented as biocontrol agents for protection against numerous soil-borne plant diseases [4–6]. However, successful biocontrol requires the formulation of carriers that ensure *Trichoderma* survival and maintenance of a significant population in soil.

Several inexpensive agricultural wastes (e.g. animal manure and agricultural industry by-products [7–9]) have been recognized as feasible organic carriers of many bio-agents. Manure addition to soil is known to improve soil fertility and positively influence crop quality and yields [10]. Bio-organic fertilizers (BOFs) that contain *Trichoderma* spp. and animal manure have been shown to promote plant growth and control Fusarium wilt in cucumber plants [5, 11, 12, 13]. In previous studies and through commercial BOF production, defatted rapeseed meal has been used as an organic nitrogen source during secondary co-solid-fermentation with manure compost for BOF production [12, 14]. While defatted rapeseed meal is used as animal feed, marginal costs for BOF production exceed marginal revenues. This opportunity cost negatively impacts the large-scale production of BOFs both in China and internationally. Therefore, there is need to develop alternative waste organic nitrogen sources to both replace defatted rapeseed meal in order to reduce BOF production costs and to identify an optimum secondary co-solid-fermentation process to ensure a maximum PGPF (*Trichoderma* spp.) population in BOFs.

Currently, animal farms in China are growing a rapid pace with average mortality rates ranging between5% and 10%. Deceased animals are typically buried or are burned, resulting in air pollution and the waste of numerous biological resources. To address this, we have developed a method to produce amino acids by hydrolyzing dead animals inH_2_SO_4_ solution (3 mol/L) at 100–105°C and 1.5–1.8 atmospheric pressure (China patent: ZL201410042218.3). The resulting amino acids are used as liquid fertilizer, as BOF additives, and have been used by several Chinese companies for the production of amino acid fertilizers.

Immature BOFs have been shown to result in negative effects on both plant growth and soil properties [15]. Therefore, maturity assessments of BOFs are necessary to guarantee effective agricultural application. Fluorescence excitation-emission matrix (EEM) spectroscopy, an efficient, inexpensive and highly sensitive technique, can be used to evaluate compost maturity levels, including the identification of protein-like, fulvic acid-like and humic acid-like substances [16]. Provenzano et al. [17] and Henderson et al. [18] used fluorescence spectroscopy as a tool to assess chemical transformations that occur during composting. However, fluorescence EEM analyses mainly depend on the visual determinations of peaks or on peak intensity ratios [19, 20], which are limited by the inherent sample heterogeneity. As a quantitative technique, fluorescence regional integration (FRI) addresses this issue and has been proven suitable for analyzing EEM spectra [21, 22].

In order to address BOF production costs, we made a first attempt to replace defatted rapeseed meal with amino acids obtained through the hydrolysis of pig corpses. The strain *Trichoderma harzianum* T-E5, a previously reported PGPF [3, 5], was utilized in the secondary solid fermentation of cattle manure compost, maggot manure and hydrolytic amino acid solution to produce a novel BOF. In addition, we aimed to identify optimized ratios of different waste proteins and matured composts to yield maximumT-E5 population levels in BOF based on RSM. Compost substance transformation was monitored using EEM and FRI to identify relationships between raw material changes and *T*. *harzianum* T-E5 growth. Lastly, we implemented a greenhouse pot experiment to determine whether the novel BOF had significant growth promotion of three different vegetable crops (cucumber, tomato and pepper).

## Materials and Methods

### Ethics statement

Our study was carried out on the field experimental station (31°20′ N, 119°40′ E)of Jiangsu Key Lab for Solid Organic Waste Utilization, Yixing, Jiangsu Province, with property rights in China (2012–2028). No specific permits were required for the described field studies and the locations were not protected. The study field did not involve endangered or protected species.

### Fungus and conidia suspension preparation

*T*. *harzianum* T-E5 (CCTCC No.AF2012011, China Center for Type Culture Collection) was used throughout the secondary solid fermentation experiment. The strain was cultured on potato dextrose agar (PDA) medium at 28°C in the dark and was stored at 4°C. *T*. *harzianum*T-E5 conidia suspension was prepared as described in Zhang et al. [3], with the final concentration of 2.9 × 10^8^ colony forming units (CFU) mL^−1^, based on hemocytometercounts.

### Raw materials

Cattle manure compost was supplied by Lianye Co., Ltd., Jiangyin City, Jiangsu Province, China, and used as the basic substrate of the secondary solid fermentation process. Maggot manure was also supplied by Lianye Co., Ltd., and rice straw was collected from a local farm. The three materials used for secondary solid fermentation were pretreated following Zhang et al. [5]. Properties of the pretreated cattle manure compost, maggot manure and rice straw samples used are shown in S1 Table. Amino acids were prepared via the acidic hydrolysis of pig corpses as follows: 3mol/L H_2_SO_4_ and pork (3:2, v/m) was mixed together in a flask (1 L); the flask containing the mixture was then heated at 100–105°C for 3 h and stirred every 30 min; the prepared hydrolytic amino acids were then stored at 4°C for later use. A chromatogram of the hydrolytic amino acids is shown in S1 Fig.

### Description of the secondary solid fermentation (composting) process

Various forms of agricultural waste (e.g. maggot manure, hydrolytic amino acids, and rice straw) and *T*. *harzianum*T-E5 conidial suspensions of various ratios were thoroughly mixed with cattle manure compost. The mixtures (3 kg) were placed into a 30 cm x 20 cm x 15 cm plastic sterile container for the second phase of solid fermentation under aerobic conditions in an open system at 40–45% and under varying ambient temperatures for 14 d. The composting piles were manually agitated daily to guarantee inoculated *T*. *harzianum*T-E5growth at the prescribed temperature. Each composting treatment contained three replicates. Samples were collected on the last day of composting, and a five-point sampling method was employed. The collected samples were immediately stored at -20°C, and *T*. *harzianum* populations in the collected samples were quantified using the Real-time Taq-Man PCR technique described by Zhang et al. [5].

### Experimental design for the optimization of *T*. *harzianum*T-E5 populations during the composting process

#### Plackket-Burman design (PBD)

Plackket-Burman design (PBD)was employed to prescreen significant factors for the *T*. *harzianum* T-E5 population from the six main variables provided at different levels: Maggot manure content (10–25%, m/m), hydrolytic amino acid content (10–20%, m/m),rice straw content (1–5%, m/m), composting temperatures (20–40°C),T-E5 inoculum content (2–10%, v/m) and daily agitation frequencies (1–3), designated as X_1_, X_2_, X_3_, X_4_, X_5_ and X_6_, respectively. Significant effects of variables on the T-E5 population were determined at 95% confidence level (*p*<0.05) based on regression analyses [23].The study experimental design and statistical data analysis were implemented using Design Expert Version 8.0.6 (State-Ease, Inc., Minneapolis, MN, USA).

#### Box-Behnken design (BBD)

Four variables, consisting of maggot manure, hydrolytic amino acid, composting temperature and *T*. *harzianum* T-E5 inoculum content levels, were selected as significant factors based on PBD and were designated as new X_1_, X_2_, X_3_ and X_4_variables, respectively. The BBD, based on response surface methodologies (RSM), was used to optimize T-E5 populations based on the four significant variables selected. Predicted responses (Y) in terms of the significant variables (X_1_, X_2_, X_3_ and X_4_) were explained using the following second-degree polynomial equation: Y = β_0_ +β_1_X_1_ +β_2_X_2_ +β_3_X_3_ +β_4_X_4_ +β_11_X_1_^2^ +β_22_X_2_^2^ +β_33_X_3_^2^ +β_44_X_4_^2^ +β_12_X_1_X_2_ +β_13_X_1_X_3_ +β_14_X_1_X_4_ +β_23_X_2_X_3_ +β_24_X_2_X_4_ +β_34_X_3_X_4_, where β_0_ is the intercept term; β_1_, β_2_, β_3_, β_4_ are the linear coefficients; β_11_, β_22_, β_33_, β_44_ are the quadratic coefficients; and where β_12_, β_13_, β_14_, β_23_, β_24_, β_34_ are the cross-coefficients. Three-dimensional (3D) response surface plots were developed to visualize independent variable interactions with the T-E5 population, and Design Expert Version 8.0.6 (State-Ease, Inc., Minneapolis, MN, USA) was employed for experimental design, regression analysis and graphical presentation purposes.

### Expanded composting under the optimized conditions

#### Compost sample collection

The expanded composting process (20 kg) under optimized conditions was conducted in triplicate and was prepared based on Section “*Description of the secondary solid fermentation (composting) process*”. Compost samples were collected daily at 4:00 PM after thorough manual stirring, and each collected sample was divided into three samples. One sample was air-dried at room temperature, and the other two were stored at 4°C and -20°C.

#### Physical and chemical analysis

The temperature of each pile at a depth of 20 cm was recorded daily at 4:00 PM over the course of the composting process. The fresh compost samples were oven dried at 105°C for ~24 h to a constant weight for moisture content determination [24]. The aqueous compost extracts [14] were obtained by mixing the fresh compost samples with deionized water (1:10, w/v) followed by horizontal shaking (160 rpm/min) for 2 h at room temperature. Extracts were then centrifuged at 10,000 rpm and filtered through 0.45 um polytetrafluoroethylene (PTFE) filters. The prepared water extracts were analyzed for pH and electrical conductivity (EC) using a pH electrode (PB-10, Sartorius, Germany) and Conductivity Indicator (LF91, Wiss Techn Werkstatten, Germany), respectively. Total carbon and nitrogen content levels were determined using an auto elemental analyzer (Vario EL III, Elementar, Germany), and NH_4_^+^-N and NO_3_^-^-N concentrations were measured using an auto analyzer (AA3, Bran and Luebbe, Germany). All physicochemical analyses of the compost samples were carried out in triplicate.

#### Fluorescence spectroscopy determination and analysis

Fluorescence spectroscopy and sample analyses were conducted following Yu et al. [16]. Water-extractable organic matter (WEOM) was prepared first by placing a mixture composed of the compost sample and deionized water (1:10, w/v) was placed on a horizontal shaker (150 rpm/min) at room temperature for 24 h. The turbid liquid was then filtered through a 0.45 μm PTFE filter following centrifugation at 10,000 rpm for 10 min. Prior to the determination of fluorescence, the WEOM filtration was diluted to a concentration of dissolved organic carbon (DOC) <10 mg/Las determined by a TOC/TN analyzer (multi N/C 3000, Analytik Jena AG, Germany).Fluorescence EEM spectroscopy measurements were performed using a fluorescence spectrophotometer (Cary Eclipse; Varian, Inc., Palo Alto, CA) in scan mode at room temperature. The conditions for obtaining fluorescence EEM spectra were as follows: a 150 W xenon excitation source; excitation wavelengths (λ_Ex_) of 200 to 500 nm at 10 nm increments; emission wavelengths (λ_Em_) of 250 to 600 nm at 2 nm increments; excitation and emission slit bandwidths of 5 nm and a scan rate of 1200 nm min^-1^. The fluorescence spectrum of deionized water sent through a 0.45 μm millipore filter under the same conditions was used as the control, and all EEM spectra results were deducted from the control. A quinine sulfate (QS) solution was used for fluorescence intensity calibration, where 0.01 mg L^-1^ quinine in 1 mol L^-1^ H_2_SO_4_ at λ_Ex_/λ_Em_ = 350/450 nm was defined as the Quinine sulfate unit (QSU) fluorescence intensity level [25]. Fluorescence EEM spectra were divided into five excitation-emission regions [26] and were then quantitatively analyzed using FRI techniques.

### Pot experiment

#### Plant materials, soils and fertilizers

Cucumber (*Cucumis sativus* L.cv. Jinchun No. 4), tomato (*Lycopersicon esculentum* L. cv. BGQF), and pepper (*Capsicum annuum*L. cv. Sujiao No. 5) seedlings were prepared following Zhang et al. [12] and Lugtenberg et al. [27]. A loamy soil was collected from a local field with no historical cucumber, tomato or pepper plants cultivation. The soil, contained 20.6 g kg^-1^of organic matter, 1.8 g kg^-1^of total N, 20.6 mg kg^-1^of available P, 124.4 mg kg^-1^of available K, 20% water content and a pH of 6.6. The cattle manure compost (CK) and organic fertilizer inoculated without T-E5 (OF) and with T-E5 (BOF) described in Section “*Expanded composting under the optimized conditions*” were used so that we could evaluate their effects on plant growth.

#### Pot experiment design

The pot experiment was performed in a greenhouse (average air temperature 30°C, relative air humidity 60%) located in the Jiangsu Key Lab for Solid Organic Waste Utilization of Yixing City in Jiangsu Province, China from May to June of 2014. The following treatments were utilized. Control, consisting of pot soil supplemented with 0.5% (w/w DW) CK and planted with cucumber (CKc), tomato (CKt) and pepper (CKp) plants, treatment 1, pot soil supplemented with 0.5% (w/w DW) OF and planted with cucumber (OFc), tomato (OFt) and pepper (OFp) plants, treatment 2, pot soil supplemented with 0.5% (w/w DW) BOF and planted with cucumber (BOFc), tomato (BOFt) and pepper (BOFp) plants. A total of 3 kg soil was placed into each plastic pot (20 × 31 cm in diameter and height). Three blocks were randomly laid out, with each treatment containing 15 replications (pots). The cucumber, tomato and pepper plants were harvested after 30 days of growth, and chlorophyll content, shoot length and plant dry weight levels were determined. The pot experiment was repeated once from July to August, 2014.

### Statistical analysis

For the composting experiment, quantification results of *Trichoderma* were analyzed using 7500 system SDS Software version 1.4 (Applied Biosystems). Sigmaplot 12.0 was employed to develop a contour map of fluorescence spectra. The Pearson correlation coefficient (R) was used to determine the linear correlation between the two parameters. As similar trends were observed for the two pot experiments, only data from May to June of 2014 are presented in this paper. Data collected included the means of the three replicates, and the statistical analyses were conducted using SPSS 13.0 (SPSS Inc., Chicago, IL, USA). Data were subjected to Duncan’s analysis of variance (ANOVA), and mean values of the different treatments were determined using Duncan’s multiple range tests at a p≤0.05 level.

## Results and Discussion

### Optimization of T-E5 populations through Plackket-Burman and Box-Behnken design experiments

The experimental design matrix and results of PBD procedure are listed in Table 1. T-E5 populations, which ranged from 10^4^ to 10^6^ ITS copies g^−1^, varied greatly across the 12 performed trials. The statistical analysis employed for the selection of significant variables is described in Table 2. The maggot manure (X_1_, p < 0.0001), hydrolytic amino acid (X_2_, p < 0.0001), composting temperature (X_4_, p < 0.0001) and inoculum content (X_5_, p = 0.0416) were identified as significant factors, whereas rice straw (X_3_, p = 0.012) and daily agitation frequency (X_6_, p = 0.30) did not have a significant effect on the T-E5 population. Fisher’s test F (1203.99) and p (< 0.0001) values showed that the overall model was statistically significant (> 99.9%) and offered excellent response result predictions. The coefficient of determination (R^2^, 0.9992 > 0.8) demonstrated good fit with the response model [28], with 99.92% of the T-E5 population sample variation explained by this model. The adjusted determination coefficient value (Adj R^2^ = 0.9985) also confirms that the actual response (T-E5 population) agreed well with the predicted response.

**Table 1:** Plackket-Burman design matrix and the results of the six variables (coded and actual levels) with T-E5 population levels as a response.

Run order	Experimental factors									Response				
	Coded level						Actual level						T-E5 population(log_10_T-E5 ITS copies g^-1^)	
	χ_1_	χ_2_	χ_3_	χ_4_	χ_5_	χ_6_	X_1_	X_2_	X_3_	X_4_	X_5_	X_6_	Actual	Predicted
1	1	1	1	-1	-1	-1	25	20	5	20	2	1	6.62	6.64
2	-1	1	1	1	-1	-1	10	20	5	40	2	1	4.95	4.96
3	-1	-1	-1	-1	-1	-1	10	10	1	20	2	1	5.89	5.90
4	-1	-1	-1	1	-1	1	10	10	1	40	2	3	4.72	4.72
5	1	-1	-1	-1	1	-1	25	10	1	20	10	1	6.46	6.45
6	-1	-1	1	-1	1	1	10	10	5	20	10	3	5.92	5.94
7	-1	1	1	-1	1	1	10	20	5	20	10	3	6.18	6.16
8	-1	1	-1	1	1	-1	10	20	1	40	10	1	5.02	5.00
9	1	1	-1	1	1	1	25	20	1	40	10	3	5.46	5.50
10	1	-1	1	1	1	-1	25	10	5	40	10	1	5.28	5.27
11	1	-1	1	1	1	-1	20	10	5	40	2	3	5.24	5.22
12	1	1	-1	-1	-1	1	25	20	1	20	2	3	6.65	6.63

Note: X_1_, maggot manure (%); X_2_, hydrolytic amino acid (%); X_3_, rice straw (%); X_4_, composting temperature (°C); X_5_, inoculum content (%); X_6_, daily agitation frequency.

**Table 2:** Analysis of variance (ANOVA) for the response surface quadratic model of the T-E5 population based on the Plackett-Burman design (PBD).

Source	T-E5 population(log_10_T-E5 ITS copies g^-1^)				
	Sum of squares (SS)	Degrees of freedom (DF)	Mean square (MS)	F Values	P-value Prob>F
Model	5.07	6	0.84	1203.99	<0.0001**
X_1_	0.77	1	0.77	1090.37	<0.0001**
X_2_	0.16	1	0.16	222.91	<0.0001**
X_3_	8.333E-006	1	8.333E-006	0.012	0.9175
X_4_	4.14	1	4.14	5902.91	<0.0001**
X_5_	5.208E-003	1	5.208E-003	7.42	0.0416*
X_6_	2.083E-004	1	2.083E-004	0.30	0.6092
Residual	3.508E-003	5	7.017E-004		
Cor Total	5.07	11			

Note: C.V. % = 0.46; R^2^ = 0.99923; Adj R^2^ = 0.9985; Pred R^2^ = 0.9960; Adeq Precision ratio = 94.655. X_1_, maggot manure (%); X_2_, hydrolytic amino acid (%); X_3_, rice straw (%); X_4_, composting temperature (°C); X_5_, inoculum content (%); X_6_, daily agitation frequency.

*Correlation is significant at *p* < 0.05

**Correlation is highly significant at *p* < 0.001.

The Box-Behnken design (BBD) approach was further employed to examine interactive effects of the four selected significant factors (maggot manure X_1_, hydrolytic amino acid X_2_, composting temperatureX_3_and inoculum content X_4_on T-E5 populations based on the PBD results. A total of 29 trials conducted with four independent variables included five center points at three levels (-1, 0, +1), and response values (from 10^5^ to 10^8^ ITS copies g^−1^) are shown in Table 3. A second-order polynomial equation was derived by conducting multiple regression analyses on the experimental data in order to explain the relationship between the independent variables and T-E5populations: Y = 8.52 +0.15X_1_ +0.06X_2_−0.48X_3_ -(4.167E-003)X_4_−0.33X_1_^2^–0.32X_2_^2^–1.98X_3_^2^–0.011X_4_^2^ -(2.5E-003)X_1_X_2_−0.04X_1_X_3_ -(2.5E-003)X_1_X_4_−0.073X_2_X_3_ +(1.0E-002)X_2_X_4_ +0.03X_3_X_4_, where Y denotes the predicted T-E5 population, and where X_1_, X_2_, X_3_ and X_4_ are the coded values for maggot manure, hydrolytic amino acid, composting temperature and inoculum content levels, respectively.

**Table 3:** Box-Behnken design (BBD) matrix of variables (coded and actual levels) for T-E5 population optimization.

Run order	Experimental factors						Response			
	Coded level				Actual level				T-E5 population (log_10_T-E5 ITS copies g^-1^)	
	χ_1_	χ_2_	χ_3_	χ_4_	X_1_	X_2_	X_3_	X_4_	Actual	Predicted
1	-1	-1	0	0	10	10	30	6	7.89	7.65
2	-1	0	0	1	10	15	30	10	7.99	8.03
3	1	0	-1	0	25	15	20	6	6.90	6.89
4	0	-1	0	1	17.5	10	30	10	8.09	8.11
5	0	0	0	0	17.5	15	30	6	8.64	8.52
6	-1	1	0	0	10	20	30	6	7.94	7.88
7	0	0	0	0	17.5	15	30	6	8.29	8.52
8	0	1	0	1	17.5	20	30	10	8.15	8.25
9	1	0	1	0	25	15	40	6	5.89	5.84
10	0	0	1	-1	17.5	15	40	2	6.13	6.02
11	0	0	1	1	17.5	15	40	10	6.15	6.07
12	-1	0	-1	0	10	15	20	6	6.34	6.50
13	0	1	1	0	17.5	20	40	6	5.74	5.72
14	0	0	0	0	17.5	15	30	6	8.57	8.52
15	0	0	-1	1	17.5	15	20	10	7.11	6.98
16	0	-1	-1	0	17.5	10	20	6	6.42	6.57
17	1	0	0	1	25	15	30	10	8.27	8.33
18	0	-1	1	0	17.5	10	40	6	5.61	5.74
19	0	0	0	0	17.5	15	30	6	8.47	8.52
20	-1	0	0	-1	10	15	30	2	7.96	8.03
21	1	1	0	0	25	20	30	6	8.09	8.08
22	0	1	0	-1	17.5	20	30	2	8.14	8.24
23	-1	0	1	0	10	15	40	6	5.49	5.62
24	0	1	-1	0	17.5	20	20	6	6.84	6.83
25	1	-1	0	0	25	10	30	6	8.05	7.97
26	0	0	-1	-1	17.5	15	20	2	7.21	7.05
27	1	0	0	-1	25	15	30	2	8.25	8.34
28	0	0	0	0	17.5	15	30	6	8.62	8.52
29	0	-1	0	-1	17.5	10	30	2	8.12	8.14

Note: X_1_, maggot manure (%); X_2_, hydrolytic amino acid (%); X_3_, composting temperature (°C); X_4_, inoculum content (%).

In this equation, the X_1_, X_2_, X_2_X_4_ and X_3_X_4_coefficients are positive, denoting that they had a synergistic effect on the T-E5 population. In contrast, the negative terms had an inverse effect on the T-E5 population [29]. Based on the p-value of this model (Table 4), we can conclude that the X_1_, X_3_, X_1_^2^, X_2_^2^, and X_3_^2^ constants had significant effects on the response results (P < 0.05) and therefore that considerable effects on the T-E5 population would emerge if minor variations between these factors were present. However, X_2_, X_4_, X_4_^2^, X_1_X_2_, X_1_X_3_, X_1_X_4_, X_2_X_3_, X_2_X_4_ and X_3_X_4_ were found to be insignificant (P > 0.05). The high significance of this model is reflected by both the F (82.19) and p value (p < 0.0001) (Table 4). The high R^2^-value (0.9880) illustrated a strong correlation between the actual and predicted results and theAdjR^2^ (0.9760) value confirmed the high significance of this model. The coefficient of variation (C.V.) was 2.15% (< 10%), implying that the designed experiment was precise and reliable. The lack of fit measure was found to be non-significant for the model (p = 0.4169), and thus the model can be deemed suitable for the prediction of variables in this case. These analytical results fully demonstrate that the response equation serves as an appropriate model for the optimization of T-E5 populations.

**Table 4:** Analysis of variance (ANOVA) for the response surface quadratic model of the T-E5 population based on the Box-Behnken design (BBD).

Source	T-E5 population(log_10_T-E5 ITS copies g^-1^)				
	Sum of squares (SS)	Degrees of freedom (DF)	Mean square (MS)	F Values	P-value Prob>F
Model	29.46	14	2.10	82.19	<0.0001**
X_1_	0.28	1	0.28	11.02	0.0051*
X_2_	0.043	1	0.043	1.69	0.2150
X_3_	2.81	1	2.81	109.87	<0.0001**
X_4_	2.083E-004	1	2.083E-004	8.137E-003	0.9294
X_1_X_2_	2.500E-005	1	2.500E-005	9.764E-004	0.9755
X_1_X_3_	6.400E-003	1	6.400E-003	0.25	0.6249
X_1_X_4_	2.500E-005	1	2.500E-005	9.764E-004	0.9755
X_2_X_3_	0.021	1	0.021	0.82	0.3802
X_2_X_4_	4.000E-004	1	4.000E-004	0.016	0.9023
X_3_X_4_	3.600E-003	1	3.600E-003	0.14	0.7133
X_1_^2^	0.69	1	0.69	26.80	0.0001**
X_2_^2^	0.68	1	0.68	26.39	0.0002**
X_3_^2^	25.40	1	25.40	992.19	<0.0001**
X_4_^2^	8.578E-004	1	8.578E-004	0.034	0.8574
Residual	0.36	14	0.026		
Lack of Fit	0.28	10	0.028	1.34	0.4169
Pure Error	0.082	4	0.021		
Cor Total	29.82	28			

Note: C.V. % = 2.15; R^2^ = 0.9880; Adj R^2^ = 0.9760; Pred R^2^ = 0.9423; Adeq Precision ratio = 25.215. X_1_, maggot manure (%); X_2_, hydrolytic amino acid (%); X_3_, composting temperature (°C); X_4_, inoculum content (%).

*Correlation is significant at *p* < 0.05

**Correlation is highly significant at *p* < 0.001.

**Fig 1:**
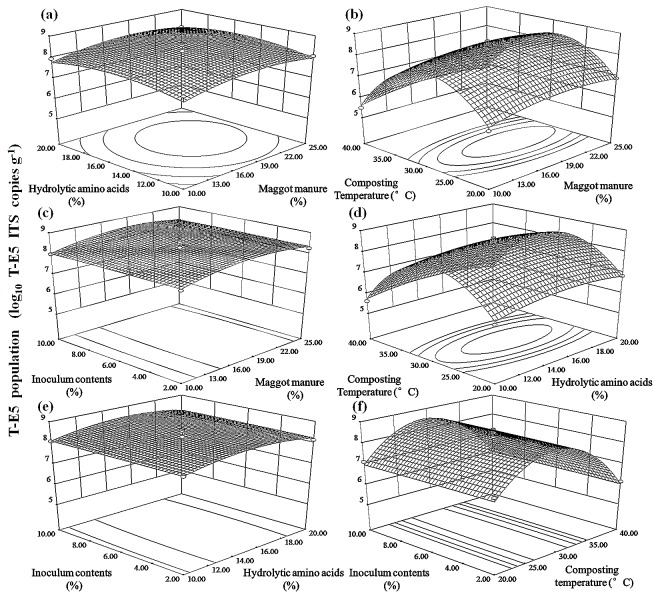
Three-dimensional response surface plot for T-E5 population as a function of maggot manure, hydrolytic amino acids, composting temperature and inoculum contents (a) maggot manure and hydrolytic amino acids; (b) maggot manure and composting temperature; (c) maggot manure and inoculum contents; (d) hydrolytic amino acids and composting temperature; (e) hydrolytic amino acids and inoculum contents; (f) composting temperature and inoculum contents.

**Fig 2:**
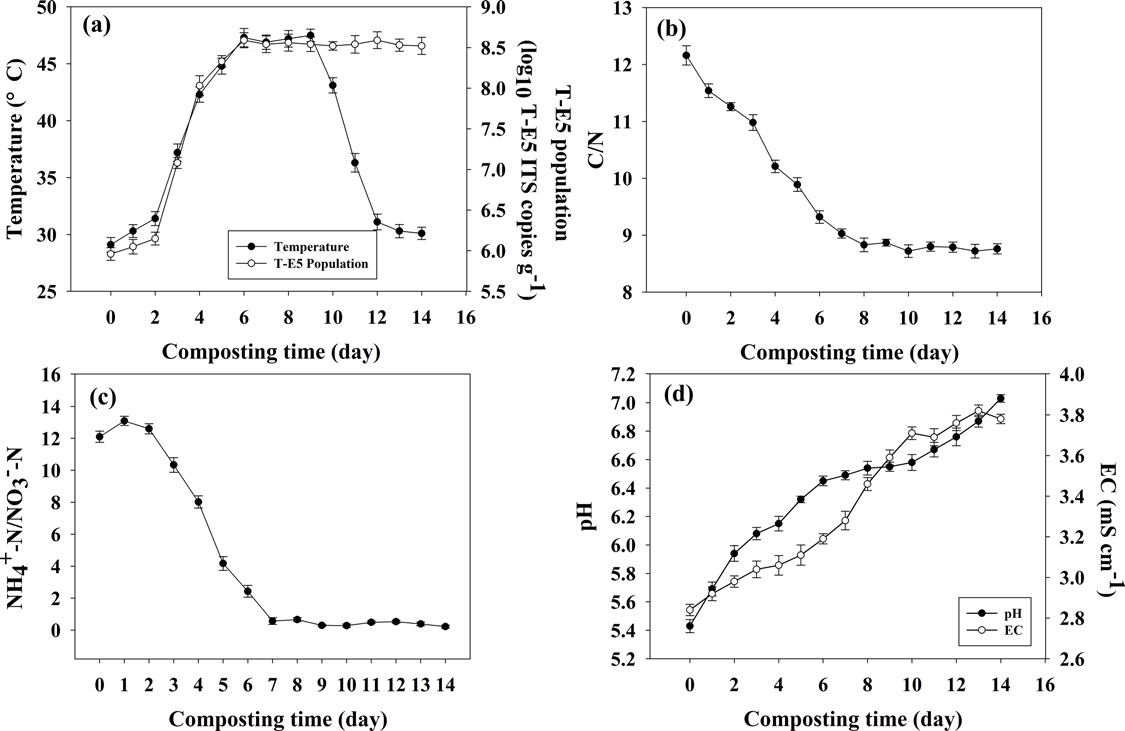
Dynamic changes of physicochemical parameters and T-E5 population during composting process in the optimized condition (a) Temperature and T-E5 population; (b) C/N;(c)NH_4_^+^-N/NO_3_^-^-N; (d) pH and EC. Note: Error bars represent standard deviation of triplicates for temperature, C/N, pH, EC, NH_4_^+^-N/NO_3_^—^N and T-E5 population.

Interactions between the variables and responses can be depicted visually by developing three-dimensional (3D) response surface plots based on the predicted model using Design-Expert software. These 3D response surface plots proved integral for locating optimal experimental conditions. Each plot (Fig 1) in our experiment was generated to illustrate the interactive effects of each pair of variables on the T-E5 population while maintaining the other two factors at moderate levels. Fig 2A shows interactions between the maggot manure and hydrolytic amino acid samples when composting temperatures and inoculum content levels were held at a value of “zero” (in our case). With an increase in maggot manure and hydrolytic amino acid levels to 17.5% and 15%, respectively, T-E5 populations increased to optimum levels (3.31 × 10^8^ ITS copies g^−1^). Thereafter, they declined despite an increase in maggot manure and hydrolytic amino acid concentrations. The analysis presented in Fig 2B-fis similar to that shown in Fig 2A. The model anticipated a maximum T-E5 population of 3.72 × 10^8^ ITS copies g^−1^ with 65.17% cattle manure compost, 19.33% maggot manure, 15.50% hydrolytic amino acid solution and 4.69% inoculum content at 28.7°C after 14 d of composting.

### Verification of the predicted optimal model

Expanded composting (20 kg) validation experiments conducted under the optimized conditions was performed in triplicate to verify the validity of the predicted results and developed model. The actual maximum average value of the T-E5 population (3.89 × 10^8^ ITS copies g^−1^) was obtained and proved to be consistent with the predicted yield (3.72 × 10^8^ ITS copies g^−1^). This strong correlation between the actual and predicted values demonstrates that this response model effectivelypredictedT-E5 population sizes.

### Physicochemical and biological parameter evolution during composting under the optimized conditions

The variations of different physicochemical properties and T-E5 population are shown in Fig 2. Temperature levels were recognized as an important parameter for characterizing compost maturity levels, and microbial activity levels were closely related to temperature changes that occurred during the composting process [30]. Temperature and T-E5 population variations at different composting stages are shown in Fig 2A. Specifically, temperatures increased from 29°C (ambient temperature) to 42°C over four days and were maintained at 42–47°C for another week. This was likely attributed to T-E5 microbial activity when labile organic substances (e.g. amino acids, soluble saccharides) were initially decomposed, followed by the slow decomposition of crop straw that maintained the temperature at 42–47°C. Temperatures then declined to less than 42°C after 10 d, suggesting compost nearing maturity. By day 13, temperatures dropped to ambient temperature levels, reflecting the consumption of available by T-E5. The T-E5 population increased rapidly until reaching its highest level by 6 d (3.89 × 10^8^ ITS copies g^−1^)and remained constant at this level (≥ 3 ×10^8^ ITS copies g^−1^) until the end of the composting phase. These dynamic changes in the T-E5 population agree well with temperatures recorded prior to compost maturity achievement (10 d).

The C/N ratio is typically used as an intuitive parameter for evaluating compost maturity levels [31]. During the composting phase, some organic C translated into CO_2_ and organic macromolecules, causing a continuous C decline. However, N content levels declined to a lesser degree (relative to C content), and thus the C/N ratio gradually declined as the composting process progressed (Fig 2B). In addition, NH_4_^+^-N/NO_3_^—^N ratios declined, in agreement with previous results [32] (Fig 2C). This result can be attributed to loss of N in the form of NH_3_in the presence of increased pH levels and compost nitrification.

The optimal range of pH during the composting stage was 5.5–8.0 [33], and compost pH levels increased with composting time. The addition of hydrolytic amino acids to the compost caused the initial compost pH (5.4) to rise rapidly after composting to 7.0 (Fig 2D), illustrating robust microbial activity levels during the composting process [20]. EC increased from 2.84 to 3.82 mS cm^-1^ (Fig 2D), attributed to the accumulation of water-soluble salt and gradually declining moisture content levels during the composting process [34]. Santamaria and Ferrera [35] demonstrated that EC reflects levels of water-soluble salt in compost and influences microbe growth and metabolism levels when EC > 8 mS cm^-1^. Thus, EC levels in our study effectively prevented the emergence of compost phytotoxicity.

### Fluorescence EEM spectra analysis for assessing compost maturity levels under optimized conditions

**Fig 3:**
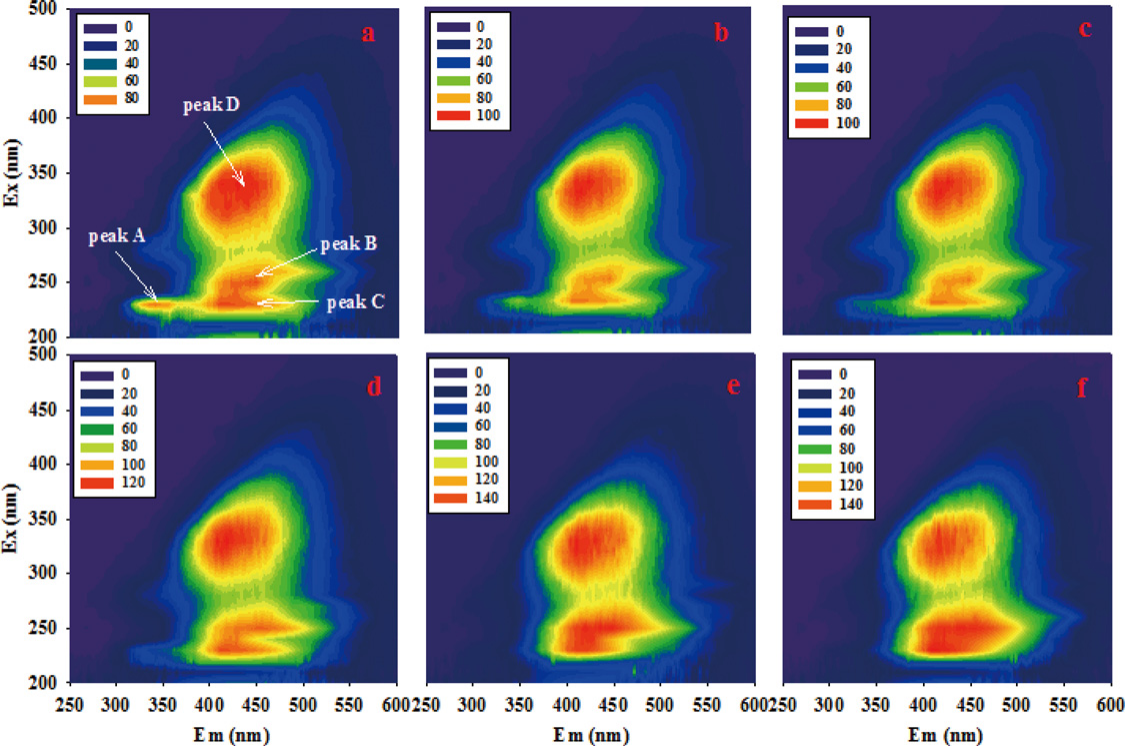
Fluorescence EEM contours of WEOM from composts during composting process in the optimized condition. Peak A, peak B and peak C represent protein-like, fulvic-like and humic-like substances, respectively. (a) 0 d; (b) 3 d; (c) 6 d; (d) 8 d; (e) 11 d; (f) 14 d.

We employed fluorescence EEM spectroscopy to monitor compost maturity in order to provide direct evidence of raw material transformation during the composting process. Water-extractable organic matter (e.g. amino acids, proteins and pigments) can emit characteristic fluorescence spectra as a result of excitation from ultraviolet or visible light and occupy specific locations along EEM fluorescence contours. Fig 3 shows the contours of WEOM that were obtained from the composting process under optimized conditions: a clear protein-like peak referred to as Peak A (λ_Ex_/λ_Em_ = 220–230 nm/300–350 nm), two fulvic-like peaks (Peak B and Peak C, λ_Ex_/λ_Em_ = 230–250 nm/400–440 nm) and a humic-like peak (Peak D, λ_Ex_/λ_Em_ = 320–340 nm/400–440 nm) were found during the initial stages of composting (Fig 3A) [26, 36]. These fluorescence EEM peaks in compost have been previously reported [17, 37, 38]. As composting progressed, Peak A grew increasingly weaker in intensity until it disappeared on day 8, while the intensity dynamics of Peaks B, C and D grew increasingly stronger (Fig 3B–3F). After the composting phase, the stability and maturity of the compost sample improved, and fulvic-like and humic-like substances became the main components of dissolved organic matter. These results are consistent with previous conclusions stating that composting was a process of the degradation of protein-like substances and the formation of fulvic-like and humic-like substances [37]. Furthermore, these results support the activity of the inoculated strain *T*. *harzianum* T-E5 (Fig 2A).

**Fig 4:**
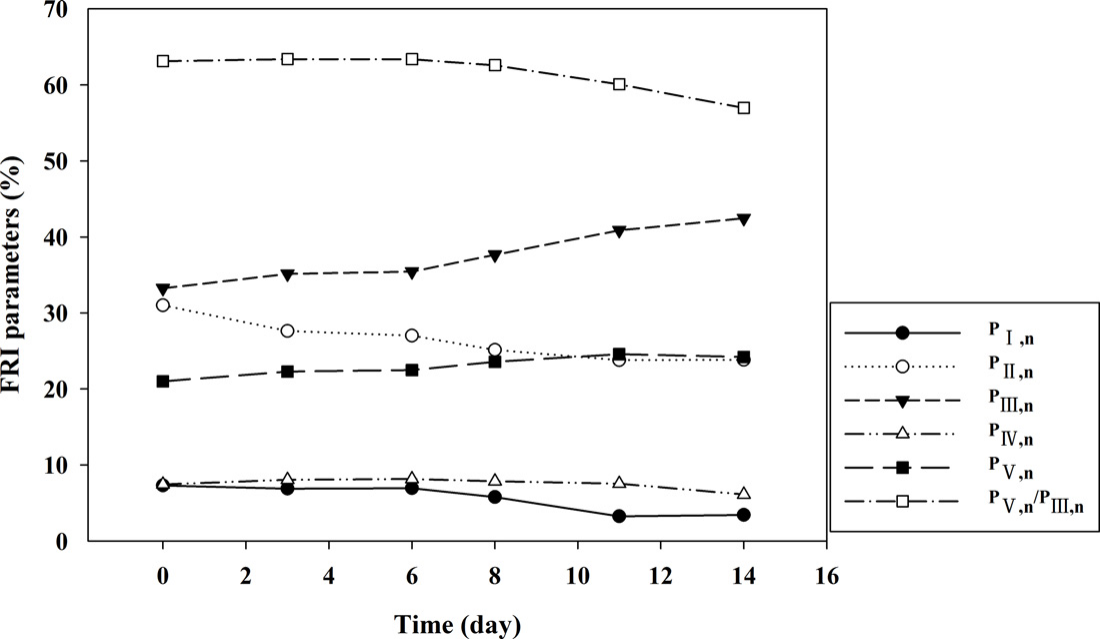
Dynamic changes of Pi, n in the five regions during composting process in the optimized condition.

The FRI technique was additionally employed for a quantitative analysis of EEM fluorescence contours. Both P_I, n_ and P_II, n_ levels decreased considerably during the first 8 days, and then held at relatively stable levels of 3.0% and 23.0%, respectively (Fig 4). This suggested that simple aromatic proteins were the main decomposition substrates in the first 8 days. In contrast, P_III, n_ and P_V, n_ levels gradually increased and reached plateau values of ~40% and ~24%, respectively, at the conclusion of the compost phase. Furthermore, P_III, n_ increased faster than that of P_V, n_. As such, P_V, n_/P_III, n_ levels declined throughout the composting process, suggesting that high molecular substances were transformed preferentially into fulvic acid-like materials, with smaller increases in humic-like substances. P_IV, n_ levels increased over the first six days and then decreased slightly, suggesting that the continued accumulation of soluble microbial products had few decomposition effects when the compost matured (after 6 d). The P_i, n_ levels observed strongly supported the above results for EEM fluorescence contours and support that composting involves protein-like substance degradation and that the formation of fulvic-like and humic-like substances occurs due to microbial activity.

A pearson correlation between the compost maturity indices and FRI parameters was conducted to investigate the potential of fluorescence EEM for evaluating compost maturity. P_I, n_ levels were found to be significantly negatively correlated with EC (R = -0.942, p < 0.01) (Table 5). P_II, n_ was found to be positively correlated with C/N (R = 0.940, p < 0.05) and NH_4_^+^-N/NO_3_^—^N (R = 0.904, p < 0.05) and significantly negatively correlated with pH (R = -0.960, p < 0.01) and EC (R = -0.965, p < 0.01). In contrast to P_II, n_, P_V, n_ was negatively correlated with C/N (R = -0.906, p < 0.05) and NH_4_^+^-N/NO_3_^—^N (R = -0.882, p < 0.05) and significantly positively correlated with pH (R = 0.918, p < 0.01) and EC (R = 0.976, p < 0.01). As with for the P_V, n_ results, positive correlations were found between P_III, n_ and the two FRI parameters (pH, R = 0.893, p < 0.05; EC, R = 0.981, p < 0.01). No significant correlation was found between the FRI parameters and P_IV, n_. The P_V, n_/P_III, n_ ratio was negatively correlated with EC (R = -0.834, p < 0.05). These results suggest that the evolution of FRI parameters (with the exception of P_IV, n_) could also be employed to characterize compost material changes and maturity.

**Table 5:** Pearson correlation between physicochemical and FRI parameters.

Parameters	P_I, n_	P_II, n_	P_III, n_	P_IV, n_	P_V, n_	P_V, n_ /P_III, n_
temperature	0.260	0.216	-0.158	0.686	0.130	0.483
C/N	0.727	0.904*	-0.802	0.291	-0.882*	0.580
NH_4_^+^-N/NO_3_^-^-N	0.716	0.940*	-0.803	0.234	-0.906*	0.552
pH	-0.789	-0.960**	0.893*	-0.431	0.918**	-0.720
Ec	-0.942**	-0.965**	0.981**	-0.565	0.976**	-0.834*

Note

*Correlation is significant at the 0.05 level (two-tailed)

**Correlation is significant at the 0.01 level (two-tailed).

### Efficacy promotion effects of produced novel *T*. *harzianum*T-E5 enhanced BOF on cucumber, tomato and pepper plant growth in the pot experiment

Significant differences were found in cucumber, tomato and pepper plant growth on the harvest day (30 days after transplantation) in the pot experiments between the control (CK) and treatment pots (OF and BOF) (Table 6). OFc (without inoculation of T-E5) exhibited significantly increased chlorophyll content, shoot lengths, and shoot and root dry weights of the cucumber plants by 12.8%, 9.6%, 18.6%, and 14.6%,respectively, as compared with the control (CKc). The addition of T-E5 enhanced organic fertilizer (BOFc)was found to enhance these responses, resulting in significant increases of 22.4%, 58.5%, 50.5%, and 34.1%, respectively. Our previous report demonstrated that T-E5 encourages cucumber plant growth via strong plant colonization and through indole acetic acid production [3]. Thus, the results of our present study show that the BOFc treatment increased chlorophyll content levels, shoot lengths, and shoot and root dry weights by 8.5%, 37.9%, 26.9%, 17.0%, respectively, relative to the OFc condition results, and that this response can be attributed to the presence of large quantities of the PGPR strain (*T*. *harzianum*T-E5) and biologically active metabolites. From the tomato and pepper plant pot experiments, similarly significant increasing tendencies as those found for the cucumber plants were found, demonstrating thatT-E5 enhanced bio-organic fertilizer has the strongest plant growth promotion effect.

**Table 6:** The effects of different treatments on the biomasses of cucumber, tomato and pepper plants on the harvest day (30 d after transplantation) during the pot experiment.

Treatments	Chlorophyll content (SPAD)	Shoot length (cm)	Dry weight (g/plant)	
			Shoot	Root
CKc	30.4±1.2c	84.6±5.0c	3.82±0.12c	0.41±0.03c
OFc	34.3±1.5b	97.2±7.0b	4.53±0.15b	0.47±0.02b
BOFc	37.2±1.2a	134.1±16.5a	5.75±0.19a	0.55±0.03a
CKt	41.5±0.9b	38.4±0.6c	4.22±0.30c	0.48±0.04b
OFt	42.4±0.7b	42.1±2.5b	4.96±0.21b	0.51±0.04b
BOFt	45.6±1.0a	47.2±1.4a	5.53±0.09a	0.63±0.05a
CKp	44.0±1.6b	33.3±2.1c	2.05±0.13c	0.33±0.03b
OFp	45.8±1.3b	37.5±0.5b	2.95±0.12b	0.35±0.02b
BOFp	49.4±1.4a	41.6±1.6a	3.79±0.13a	0.41±0.03a

Notes: Control, the pot soil was supplemented with 2% (w/w DW) of cattle manure compost and was planted with cucumber (CKc), tomato (CKt) and pepper (CKp) plants; treatment 1, the pot soil was supplemented with 2% (w/w DW) of OF and was planted with cucumber (OFc), tomato (OFt) and pepper (OFp) plants; treatment 2, the pot soil was supplemented with 2% (w/w DW) of BOF and was planted with cucumber (BOFc), tomato (BOFt) and pepper (BOFp) plants. The data were analyzed using Duncan’s ANOVA test. All values are the means of the three replicates. Values with a different letter shown in the same column are significantly different at P≤0.05. Numbers followed by “±” are the standard errors (SEs).

## Conclusions

We used hydrolytic amino acids derived from pig corpses to reduce BOF production, and developed an operational means for increasing *T*. *harzianum* T-E5 densities in BOF products through the use of various agricultural wastes. The resulting BOF can be used as a soil amendment to substantially facilitate plant growth in agricultural practices. The fluorescence EEM spectroscopy and FRI analysis results serve as a theoretical basis for developing systematic compost maturity assessment methods to ensure the secure application of BOFs in the field.

## Supporting Information

S1 Fig The chromatogram of hydrolytic amino acids (100-fold diluted solution). (DOCX)

S1 Table The basic characteristics of three agro-industrial wastes. (DOCX)
