# Supplementary figures and images for: Hydrolytic Amino Acids Employed as a Novel Organic Nitrogen Source for the Preparation of PGPF-Containing Bio-Organic Fertilizer for Plant Growth Promotion and Characterization of Substance Transformation during BOF Production
Source: PLoS One. 2016 Mar 14;11(3):e0149447. doi: 10.1371/journal.pone.0149447 (PMC4790899; doi:10.1371/journal.pone.0149447)

**S1 Fig.** The chromatogram of hydrolytic amino acids (100-fold diluted solution)


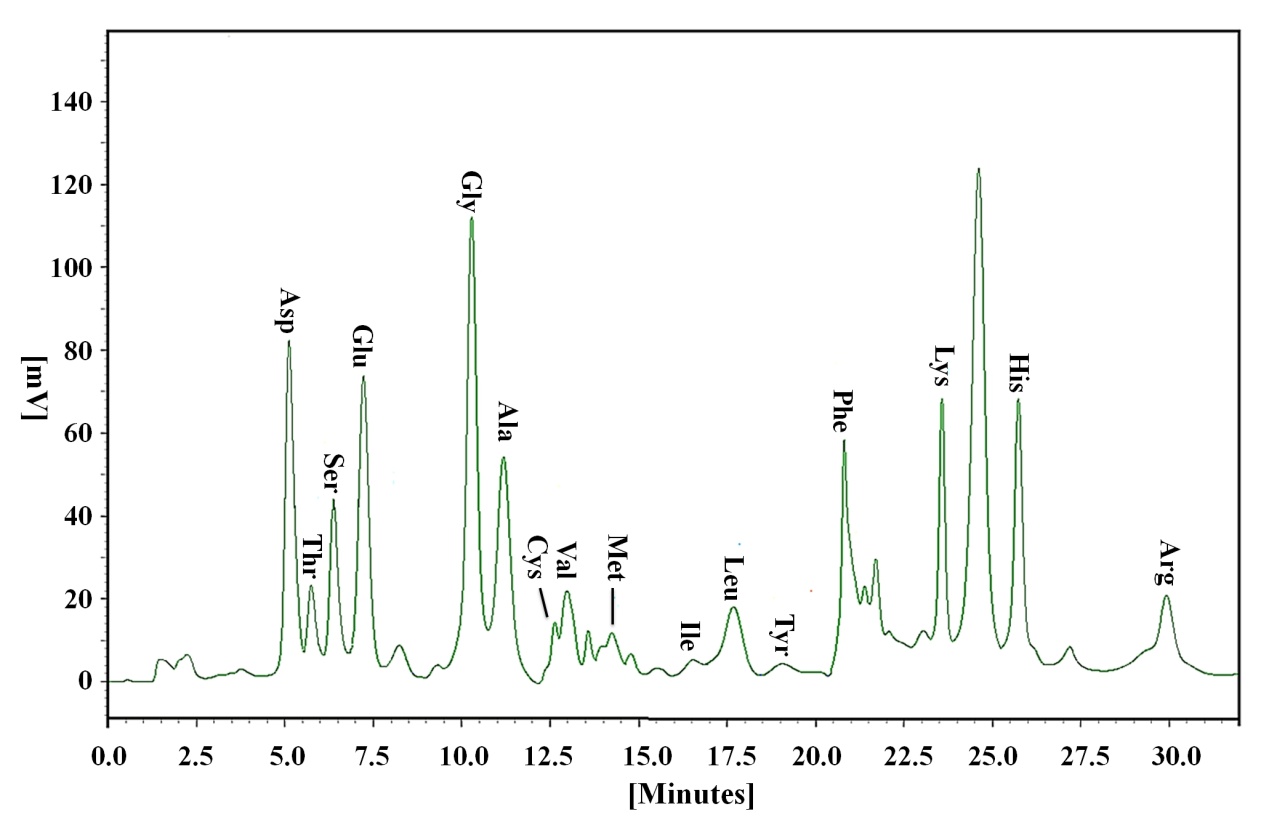


**S1 Fig.**

Supplement: S1 Fig — (DOCX) [file pone.0149447.s001.docx]
