# Supplementary material for: Hydrolytic Amino Acids Employed as a Novel Organic Nitrogen Source for the Preparation of PGPF-Containing Bio-Organic Fertilizer for Plant Growth Promotion and Characterization of Substance Transformation during BOF Production
Source: PLoS One. 2016 Mar 14;11(3):e0149447. doi: 10.1371/journal.pone.0149447 (PMC4790899; doi:10.1371/journal.pone.0149447)
Supplement: S1 Table — (DOCX) [file pone.0149447.s002.docx]

**S1 Table** The basic characteristics of three agro-industrial wastes

| Materials | Moisture  (%) | Total carbon  (%) | Total nitrogen  (%) | Carbon to nitrogen ratio (C/N) |
| --- | --- | --- | --- | --- |
| Cattle manure | 27.93 | 18.95 | 1.31 | 14.47 |
| Maggot manure | 25.02 | 19.94 | 2.09 | 9.54 |
| Rice straw | 4.98 | 47.37 | 1.12 | 42.29 |
